# Impact of alternating electric fields therapy for newly diagnosed WHO grade 4 astrocytoma on patient survival: a real-world propensity-score adjusted prospective multicenter study

**DOI:** 10.1007/s11060-025-04985-3

**Published:** 2025-03-11

**Authors:** Peter Y. M. Woo, Jenny K. S. Pu, Lai-Fung Li, Desiree K. K. Wong, Victor K. H. Hui, Danny T. M. Chan, Michael W. Y. Lee, Tony K. T. Chan, Jason M. K. Ho, Ka-Man Cheung, Teresa P. K. Tse, Sarah S. N. Lau, Joyce S. W. Chow, Natalie M. W. Ko, Herbert H. F. Loong, Aya El-Helali, Tai-Chung Lam, Fung-Ching Cheung, Wai-Sang Poon

**Affiliations:** 1 Department of Neurosurgery, Prince of Wales Hospital, 30-32 Ngan Shing Street, Shatin, N.T Hong Kong; 2 Department of Neurosurgery, Queen Mary Hospital, Hong Kong Island, Hong Kong; 3 Department of Neurosurgery, Pamela Youde Nethersole Eastern Hospital, Chai Wan, Hong Kong; 4 Department of Neurosurgery, Princess Margaret Hospital, Kwai Chung, Hong Kong; 5 Department of Neurosurgery, Tuen Mun Hospital, Tuen Mun, Hong Kong; 6 Department of Clinical Oncology, Queen Elizabeth Hospital, Kowloon, Hong Kong; 7 Department of Neurosurgery, Queen Elizabeth Hospital, Kowloon, Hong Kong; 8 Department of Neurosurgery, Kwong Wah Hospital, Kowloon, Hong Kong; 9 Department of Clinical Oncology, The Chinese University of Hong Kong, Shatin, N.T., Hong Kong; 10 Department of Clinical Oncology, The University of Hong Kong, Hong Kong Island, Hong Kong

**Keywords:** Alternating electric fields, Tumor treating fields, WHO grade 4 astrocytoma, Glioblastoma, Temozolomide chemoradiotherapy, Overall survival

## Abstract

**Purpose:**

Alternating electric fields (AEF) therapy in addition to temozolomide chemoradiotherapy (TMZ CRT) is increasingly being recommended as first-line treatment for patients with newly-diagnosed WHO grade 4 astrocytoma. However, few have validated this treatment with real-world evidence.

**Methods:**

Consecutive adult patients with newly-diagnosed WHO grade 4 astrocytoma treated with adjuvant TMZ CRT across all neuro-oncology centers in Hong Kong were reviewed. Identified from a territory-wide prospective glioma registry, propensity-score matching (1:2) was performed to match patients that either received TMZ CRT with AEF or TMZ CRT alone. Matching was according to age, Karnofsky performance status, *IDH-1* mutation, p*MGMT* methylation and extent of resection. The primary endpoint was overall survival (OS). Secondary endpoints were the incidence of AEF-associated adverse effects and mean monthly treatment compliance.

**Results:**

141 patients were reviewed, of whom 47 patients received AEF with TMZ CRT and 94 had CRT alone. Multivariate Cox proportional hazards analysis revealed that patients with p*MGMT-*methylated tumors (mOS: 30.8 months vs. 16.7 months [95% CI: 1.9–4.7] and those that received AEF (mOS: 22.8 vs. 14.3 months [95% CI: 1.9–4.7]) had longer OS. AEF therapy patients had a mOS benefit of 8.5 months. The mean monthly treatment compliance was 74 *±* 12%. A compliance threshold of 60% conferred a survival benefit of 4.1 months (mOS: 21.5 months vs. 17.4 months [95% CI: 0.10–0.96]). The only identified AEF-associated adverse reaction was scalp dermatitis that occured in 77% (36/47) of patients.

**Conclusion:**

This post-approval study offers real-world evidence in support of the use of AEF therapy as first-line treatment.

## Introduction

World Health Organisation (WHO) grade 4 astrocytoma is the commonest primary malignant tumor in adults with a prevalence of 1–5 per 100 000 population [1, 2]. In spite of standard-of-care (SOC) multimodality treatment, comprising of maximal safe resection and temozolomide chemoradiotherapy (TMZ CRT), the median overall survival (mOS) remains only 11–15 months [1, 3]. Since 2005 there has been no breakthrough treatment that has resulted in a significant and consistent improvement in OS.

Alternating electric fields (AEF), otherwise known as tumor-treating fields, is a novel therapy that involves the application of non-invasive transcranial regional AEF of low intensity (1–3 V/cm) and intermediate frequency (200 kHz) to the post-resection cavity through the placement of scalp transducer arrays [4]. Preclinical studies observed that the application of these electric fields resulted in tumor cell mitotic arrest by dielectrophorectic disruption of spindle formation during the metaphase [4, 5]. Its clinical efficacy was supported by the landmark EF-14 phase III randomized-controlled trial (RCT) that demonstrated a significant increase in mOS among newly-diagnosed glioblastoma patients that received AEF in addition to TMZ CRT compared to those that received CRT alone (21 *versus* 17 months; 95% CI: 0.53–0.76) [6]. These findings were corroborated by several smaller scale single-arm prospective or case-controlled studies as well as two meta-analyses [7–19]. However, there is a relative lack of evidence derived from real-world experience analyzing prospectively collected data comparing a meticulously selected control group that accounted for widely acknowledged survival prognostic factors such as age, Karnofsky performance status (KPS), O [6]-methylguanine-methyl transferase (p*MGMT*) promoter methylation status and tumor extent of rection (EOR).

There are several challenges of translating RCT findings to real-world practice, particularly for glioblastoma. They can be broadly classified into issues related to the complexities of oncobiology such as tumoral genetic, epigenetic, transcriptomic or microenvironment heterogeneity, the limitations of primary SOC treatment and the difficulties posed by existing clinical trial designs. Inter- and intratumoral heterogeneity indicate how variability between individuals and within different regions of the same tumor complicates the identification of a single predictive biomarker or therapeutic target thereby increasing the likelihood of treatment failure beyond the highly-controlled context of a trial [20]. For real-world cohorts, a substantial proportion of patients would be excluded from RCTs due to the inadequate effectiveness of first-line TMZ CRT, whereby either 59% progressed during therapy and 63% failed to complete it [21]. Consequently, the majority of patients encountered in daily neuro-oncological practice would not meet fundamental eligibility criteria for interventional trials. Finally, most RCTs focus on a highly selected subgroup of patients with a favorable prognostic risk profile and fail to stratify for subjects with unfavorable clinical features for example, older then 70 years or have poorer functional performance. This is one of the major reasons why epidemiological studies generally document appreciably shorter patient OS than described in RCTs [1, 22]. 

Real-world evidence studies offers a more decisive external validation on the effectiveness of novel therapies without the need to commit to the intensive resource demands of a clinical trial. It is therefore crucial to identify appropriate comparison control group patients in order to attain meaningful conclusions. To achieve this, a propensity-score matched multi-center study derived from prospectively collected glioma registry data was performed.

## Materials and methods

### Study population and data collection

This was an investigator-initiated multicenter study that analyzed prospectively collected data of propensity-score matched WHO grade 4 astrocytoma patients that either received AEF with TMZ CRT or CRT alone. The study was approved by the Hong Kong Hospital Authority (HA) institutional review board (reference number: UW 19–626) and was conducted according to the Declaration of Helsinki and Good Clinical Practice. Hong Kong is a special administrative region in China with a population of 7.8 million where 94% of the population is ethnic Chinese [23]. Universal healthcare is delivered by the HA, a statutory body that manages all public hospitals, responsible for 90% of inpatient bed-days in the city. Consecutive adult patients (*≥* 18 years-old) from all of the city’s seven neuro-oncology centers with newly diagnosed, histologically-confirmed WHO grade 4 astrocytoma from 1 January 2009 to 30 June 2022 were reviewed [24]. The diagnosis was made in accordance to the 4th WHO Classification of Tumors of the Central Nervous System (CNS) and all subjects completed TMZ chemoradiotherapy [25]. The standard treatment dose for TMZ chemotherapy was 75mg/m^2^/day for six weeks and was prescribed concomitantly with radiotherapy of 60 Gy over 30 fractions [3]. Subsequent maintenance chemotherapy comprised of TMZ 150-200mg/m^2^/day for five days every four weeks for at least six cycles was administered [3]. AEF therapy (Optune™, Novocure GmbH, Root, Switzerland) was first introduced to Hong Kong in January 2019 where patients either self-financed their treatment or were fully subsidized via the HA AEF pilot scheme, a service where selected patients of *≤* 70 years were offered the treatment free-of-charge [26]. AEF was administered within seven weeks after CRT in accordance to the EF-14 study and patients were encouraged to comply to treatment for *≥* 18 h a day or achieve a mean monthly device usage of *≥* 75%.^6^ All patients were clinically assessed at one-to-three monthly intervals with regular MRI scanning performed every three-to-six months. Patients that developed progressive disease before or during the concomitant CRT phase, could not complete CRT, only underwent a tumor biopsy, had the tumor located in the cerebellum, received prior radiotherapy, had unknown tumor isocitrate dehydrodegenase-1 (*IDH-1*) mutation status, unknown p*MGMT* methylation status, had a prior histopathological diagnosis of a lower grade glioma or had a concomitant disabling condition that precluded a preoperative KPS of *≥* 80 were excluded. Patients were not considered candidates for AEF if they experienced an active scalp or CNS infection, medically refractory seizures, radiotherapy-induced skin toxicity of Radiation Therapy Oncology Group (RTOG) grade 4, had a deep brain stimulation implant or had inadequate caregiver support. All AEF subjects or their legal representatives provided written informed consent.

Clinical data was retrieved from the Hong Kong Glioma Registry, a prospectively-collected population-level central database of adult patients with histologically-confirmed glioma from 2010 to 2022 [1]. Data was categorized into patient-, tumor- and treatment-related factors. Patient-related data included age, gender, preoperative KPS and post-concomitant CRT KPS. Post-concomitant CRT KPS was selected for functional performance assessment as AEF would be initiated at this time point. Tumor-related data included its location, *IDH-1* mutation status and p*MGMT* methylation status. *IDH-1* mutations were either determined by immunohistochemistry or by DNA sequencing if the former results were equivocal or if the patient was younger than 55 years-old. p*MGMT* methylation was ascertained by methylation-specific polymerase chain reaction testing. EOR was determined either by reviewing postoperative day-one magnetic resonance imaging (MRI) gadolinium contrast-enhanced scans on workstations installed with Centricity Enterprise Web (General Electric Medical Systems, Barrington, Illinois, USA) image viewers or when such scans were not available, by the neurosurgeon’s assessment documented in the operation records. EOR was categorized in accordance with the Response Assessment in Neuro-oncology (RANO) *resect* group criteria [27]. The postoperative MRI presence of residual tumor after the concomitant phase of CRT was also documented. The use of regional treatments such as interstitial chemotherapy, laser interstitial thermal therapy or intracavitary radiotherapy was determined. The primary endpoint was OS, defined as the duration from the date of the first surgery that confirmed the diagnosis of WHO grade 4 astrocytoma until death. The secondary endpoints were progression-free survival (PFS), mean monthly AEF therapy compliance, i.e. device usage, and its associated adverse effects. PFS was defined as the duration from the date of the first surgery to the date of clinical and/ or radiological progression. All cases were censored by 30 September 2023.

### Statistical analysis

Patients that received AEF + CRT were matched with patients from the CRT alone group using the *matchit* package in R (version 4.1.0) [28]. A propensity score was estimated using a fitted logistic regression model to predict the probability of receiving AEF therapy upon completion of CRT founded on a set of covariates [29]. These a priori factors were: gender, age, preoperative KPS (80–100 vs. < 80), post-concomitant CRT KPS (80–100 vs. < 80), tumor location by lobe, tumor location by hemisphere, *IDH-1* mutation (wildtype vs. mutant), p*MGMT* methylation (methylated vs. unmethylated), EOR and residual tumor after concomitant CRT. For the propensity score model, a linear relationship between continuous covariates and the log-odds of receiving AEF therapy were assumed. The *matchit* package employed a nearest neighbour matching algorithm to form a 1:2 ratio propensity score-matched study sample of AEF-treated to CRT alone patients using a logit calliper width of 0.2 of the standard deviation [30]. 

Demographic cohort data was summarized using standard descriptive statistics. To test differences between the groups, the Pearson’s chi-squared test (categorical variables), two-tailed Student’s t-test for independent groups (continuous variables) and one-way analysis of variance (ANOVA) was carried out for continuous variables with more than two groups. Survival analysis was performed using multivariate Cox proportional hazards modelling. Survival probabilities were represented by Kaplan-Meier plots and subgroup analysis by log-rank testing. *Post-hoc* sensitivity analysis was performed to compare OS between CRT-alone control group patients and those that were not selected for propensity score matching. A *p*-value of < 0.05 was considered statistically significant. These tests were performed utilizing the Statistical Package for the Social Sciences software version 21.0 (SPSS Inc., Chicago, Illinois, USA).

## Results

**Fig. 1:**
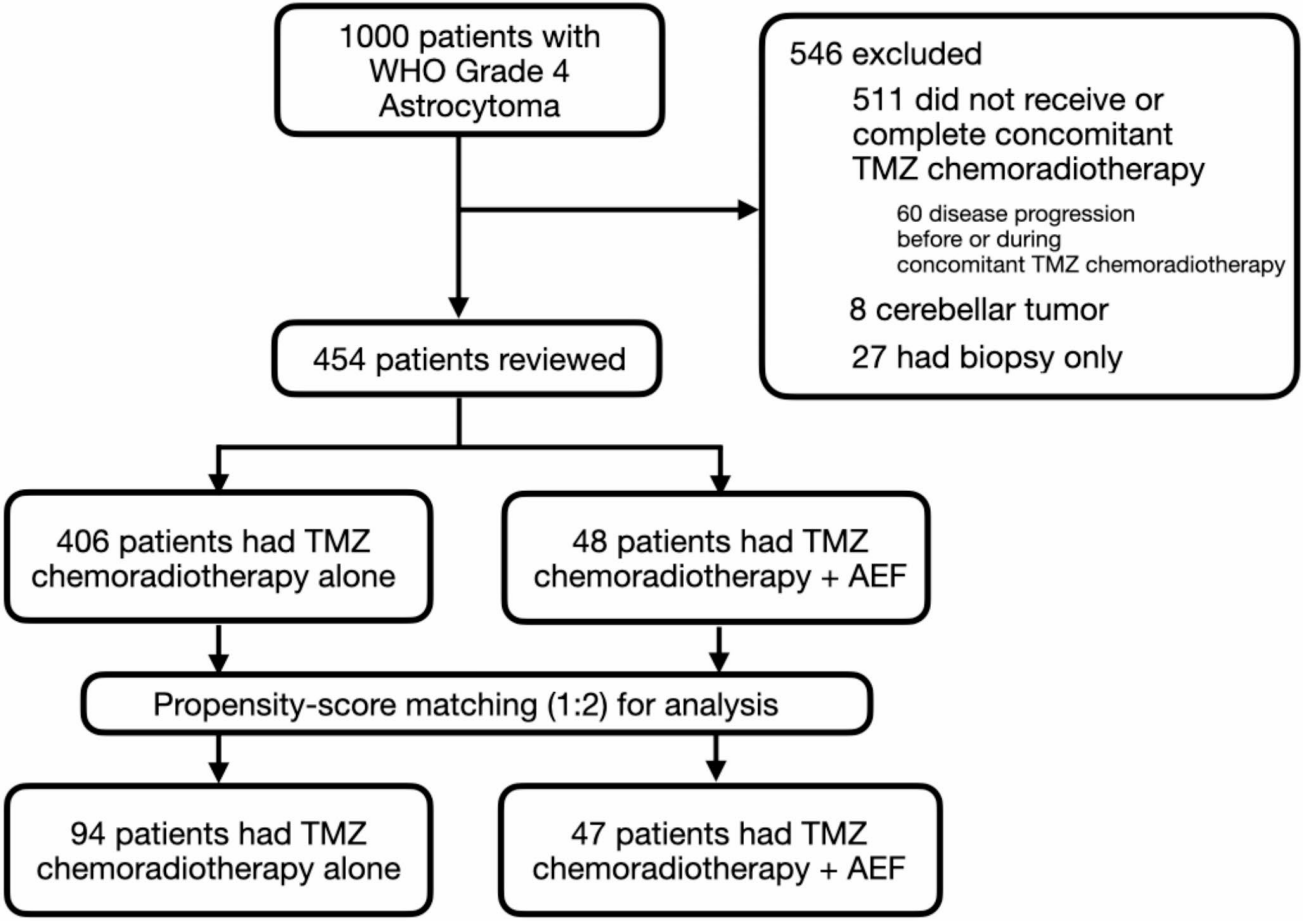
Flowchart of the Hong Kong Glioma Registry patients diagnosed with WHO grade 4 astrocytoma that were reviewed and final selection for analysis after propensity-score matching. N. B. TMZ, temozolomide; AEF, alternating electric fields

During this 14-year period, 1000 patients with histologically-confirmed newly-diagnosed WHO 4 astrocytoma were screened and 454 (45%) were eligible for review (Fig. 1). All patients were ethnic Chinese patients. 48 (11%, 48/454) patients received AEF in addition to TMZ CRT. After a 1:2 propensity score adjustment, a cohort of 141 patients comprising 47 AEF + CRT patients and 94 CRT control patients were identified (Table 1). The mean follow-up duration for the entire cohort was 26.5 *±* 14.9 months. The mean age of was 52 *±* 13 years old (range: 18–78) with a female: male ratio of 1:2. Most patients had a preoperative and post-concomitant CRT KPS of *≥* 80, i.e. 74% (104/141) and 73% (103/141) respectively. There was neither a significant difference in functional performance for the entire cohort (*p*-value: 0.82) nor among the CRT-alone and AEF + CRT groups (*p*-value: 0.77). Most tumors were located in the temporal lobe (38%, 54) followed by the frontal (33%, 47) and parietal lobes (14%, 19). 9% of tumors (13/141) were *IDH-1* mutant and 44% (62) were p*MGMT* methylated. Supramaximal (i.e. RANO class 1) or complete contrast-enhancing lesion resection (i.e. RANO class 2A) was achieved in 43% (60) of patients. Post-concomitant CRT MRI scans were performed at a mean duration of 97 *±* 23 days after diagnosis and residual contrast-enhancing tumor was detected in 52% (73) of patients. None of the patients received regional therapy such as interstitial chemotherapy, intracavitary radiotherapy or laser interstitial thermal therapy as first-line treatment. None of the patients were recruited in a clinical intervention trial. Apart from preoperative KPS and post-concomitant CRT KPS, no significant difference with regard to conventional prognostic factors were observed between the AEF + CRT and CRT control groups (Table 1). After propensity-score matching, patients in the AEF + CRT and CRT groups were comparable in all matched subgroups in terms of patient-, tumor-related factors, EOR and residual tumor after concomitant CRT.

**Table 1:** Patient characteristics of the unmatched and matched overall cohorts

	**Unmatched Cohorts**		*P*-value	Matched Cohorts		*P*-value
	Control Group	**Intervention Group**		Control Group	**Intervention Group**	
	TMZ CRT Alone	TMZ AEF + CRT		TMZ CRT Alone	TMZ AEF + CRT	
	**n = 406 (%)**	**n = 48 (%)**		**n = 94 (%)**	**n = 47 (%)**	
**Patient-factors**						
Gender						
Male	252 (62)	29 (60)	0.824	67 (71)	28 (60)	0.162
Age at diagnosis, years,						
Mean ± SD	55 ± 13	53 ± 13	0.637	53 ± 13	54 ± 13	0.669
≥ 65 years	83 (20)	7 (15)	0.336	13 (14)	7 (15)	0.864
Range	18–81	23–76	-	19–78	23–76	-
Preoperative KPS						
≥ 80	206 (51)	35 (73)	0.004	70 (70)	34 (72)	0.793
Post-concomitant CRT KPS						
≥ 80	197 (49)	34 (71)	0.003	69 (73)	34 (72)	0.833
**Tumor-factors**						
Location						
Frontal	149 (37)	18 (38)	0.542	30 (32)	17 (36)	*0.812*
Temporal	115 (28)	17 (35)	0.507	37 (39)	17 (36)	*0.75*
Parietal	102 (25)	7 (15)	0.062	12 (13)	7 (15)	*0.734*
Occipital	22 (5)	3 (6)	0.824	6 (6)	3 (6)	*0.945*
Insula	18 (4)	3 (6)	0.948	9 (10)	3 (6)	*0.588*
Laterality						
Left hemisphere	184 (45)	25 (52)	0.407	38 (40)	24 (51)	0.223
*IDH-1* mutant	28 (7)	5 (10)	0.628	8 (9)	5 (11)	0.7
*pMGMT* methylated	160 (40)	20 (44)	0.522	42 (45)	20 (43)	0.881
**Treatment-factors**						
Extent of resection*						
Class 1:						
supramaximal CE resection	65 (16)	9 (19)	0.758	14 (15)	6 (13)	0.801
i.e. residual tumor:						
0cm^3^ CE + ≤ 5cm^3^ nCE						
Class 2						
maximal CE resection	104 (26)	12 (26)	0.893	25 (27)	15 (32)	0.551
A: complete CE resection						
i.e. residual tumor:						
0cm^3^ CE + > 5cm^3^ nCE						
B: near total CE resection	227 (56)	27 (56)	0.912	55 (59)	26 (55)	0.733
i.e. residual tumor:						
≤ 1 cm^3^ CE						
Class 3						
submaximal resection	10 (2)	0	0.944	0	0	-
A: subtotal CE resection:						
i.e. residual tumor:						
≤ 5cm^3^ CE						
B: partial CE resection:	0	0	-	0	0	-
i.e. residual tumor:						
> 5cm^3^ CE						
Class 4:						
biopsy	0	0	-	0	0	-
no tumor volume reduction						
Post-concomitant CRT residual tumor	207 (51)	27 (56)	0.803	47 (50)	26 (55)	0.878

N.B. TMZ, temozolomide; CRT, chemoradiotherapy; AEF, alternating electric fields; KPS, Karnofsky performance status, GTR, gross total resection; STR, subtotal resection; *IDH-1*, isocitrate dehydrogenase-1; p*MGMT*, promoter region of methylguanine-methyltransferase; CE, contrast-enhancing; nCE, non-contrast-enhancing

*Absolute residual tumor volume according to RANO *resect* criteria by Karschnia et al. (in *Journal of Neuro-oncology*, 2023)

### Predictors for survival

**Fig. 2:**
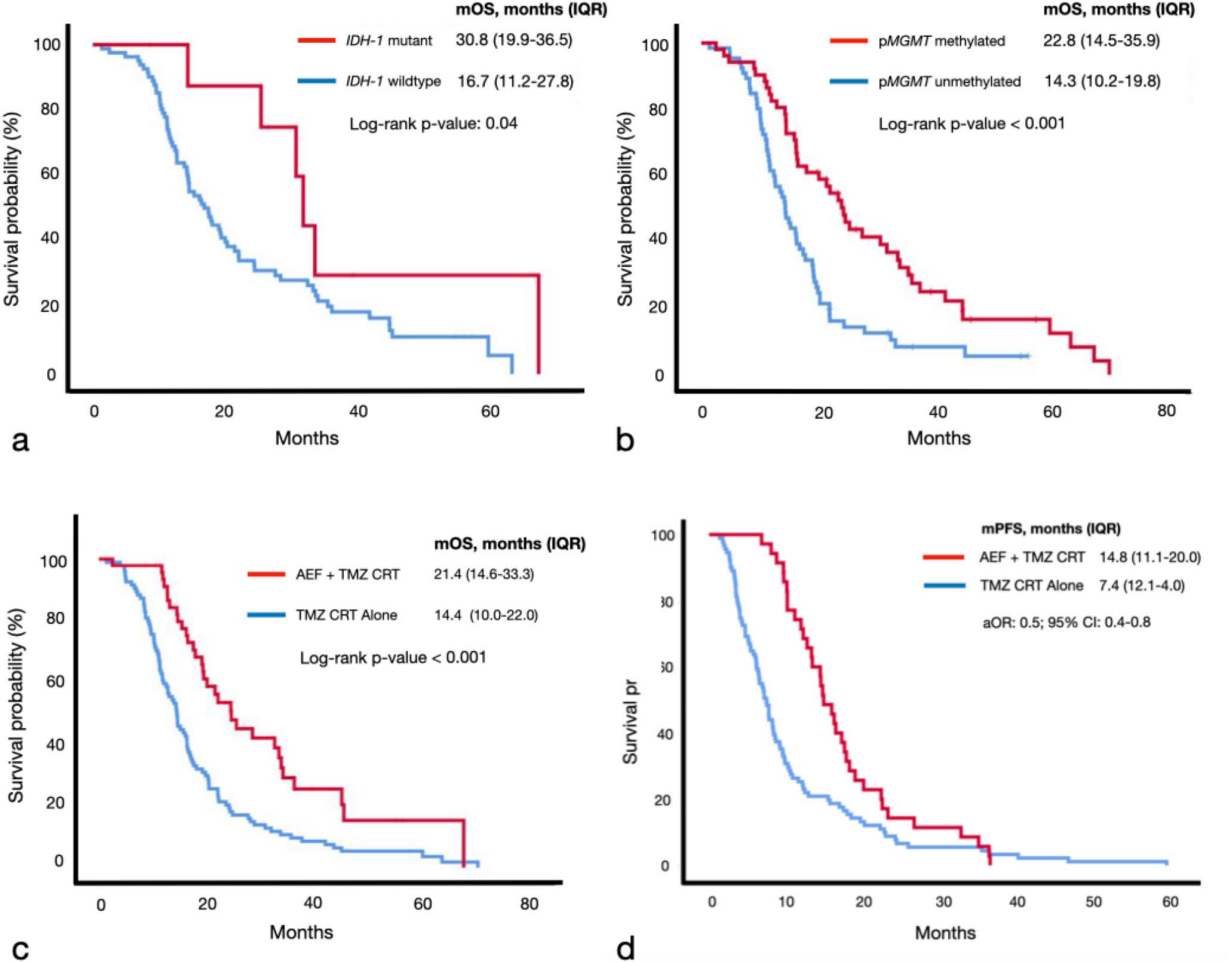
Kaplan-Meier survival analysis demonstrating predictors for overall survival for the entire patient cohort (**a-c**) and the effect of AEF on progression-free survival (**d**). N.B. TMZ, temozolomide; CRT, chemoradiotherapy; AEF, alternating electric fields; *IDH-1*, isocitrate dehydrogenase-1; p*MGMT*, promoter region of methylguanine-methyltransferase

**Fig. 3:**
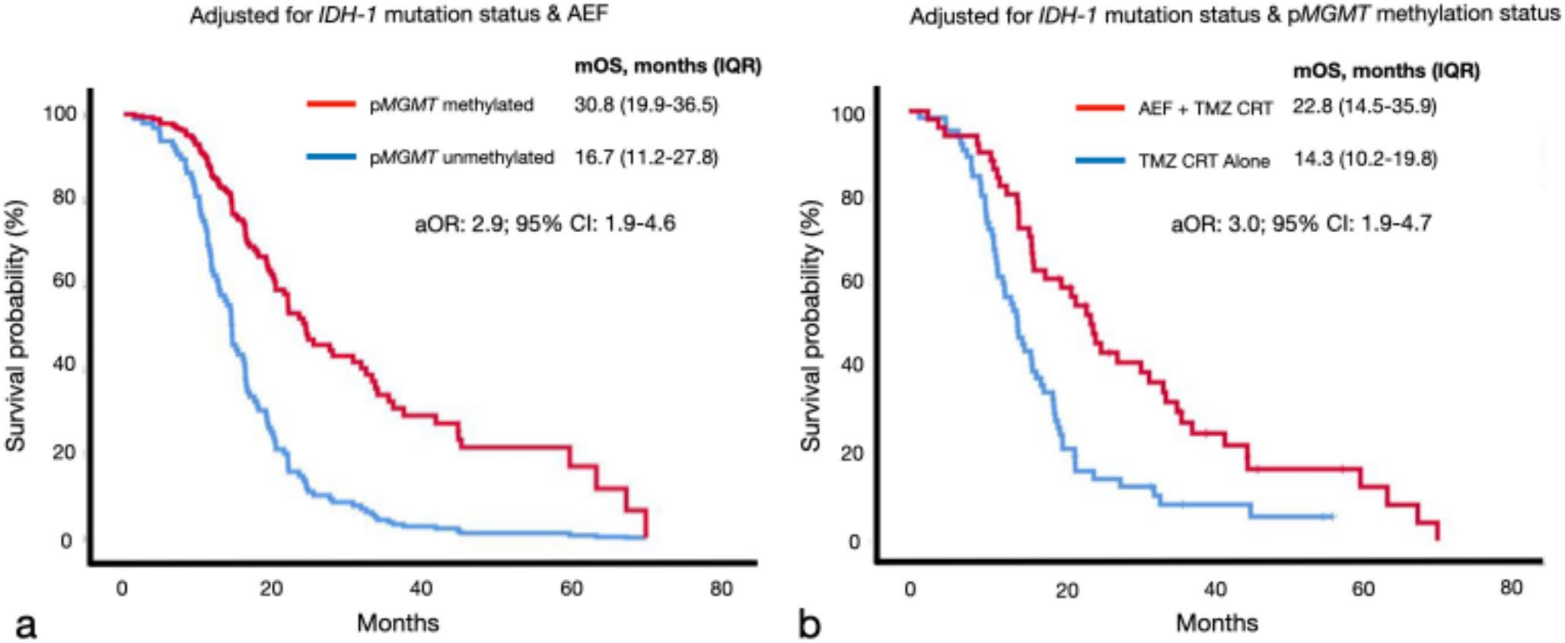
Kaplan-Meier survival analysis of independent predictors for overall survival. N.B. TMZ, temozolomide; CRT, chemoradiotherapy; AEF, alternating electric fields; p*MGMT*, promoter region of methylguanine-methyltransferase

Overall, the mOS was 16.3 months (IQR: 11.2–24.5) with the proportion of patients achieving 12-, 18- and 24-month survival being 68% (96/141), 42% (59) and 26% (36) respectively. From univariate analysis, predictors for improved OS were: *IDH-1* mutant tumors (log-tank test, *p*-value: 0.04), p*MGMT* methylated tumors (*p*-value < 0.001) and AEF (*p*-value < 0.001) (Fig. 2). Patients that received AEF had a mOS survival benefit of 7.0 months compared to those that did not (21.4 months *versus* 14.4 months). Multivariate Cox proportional hazards analysis revealed that p*MGMT-*methylated tumors (adjusted OR: 4.0; 95% CI: 2.1–7.4) and patients that received AEF (aOR: 3.8; 95% CI: 2.2–6.6) were independent predictors for OS (Fig. 3). After adjusting for confounding factors, patients with p*MGMT*-methylated tumors had a mOS benefit of 14.1 months (log-rank test, *p*-value < 0.001) and those that received AEF had an improved mOS of 8.5 months (22.8 months *versus* 14.3 months, *p*-value < 0.001). Multivariate binary logistic regression for 12-, 18- and 24-month survival revealed that AEF was an independent treatment factor (Table 2). The odds for AEF + CRT patients to reach these survival times points was fourfold greater than those that only received CRT. The 12-, 18- and 24-month survival rates of patients that received AEF were 85% (40/47), 62% (29) and 40% (19) respectively. In contrast, patients that received CRT alone had corresponding survival rates of 60% (56/94), 32% (30) and 18% (17). For the entire cohort the median PFS was 9.6 months (IQR: 5.4–16.5). Kaplan-Meier survival analysis revealed that AEF + CRT patients had a significantly longer median PFS compared to those that only received CRT (log-rank test, *p*-value: 0.003) (Fig. 2).

**Table 2:** Predictors for 12-month, 18-month and 24-month overall survival

	12-month OS		18-month OS		24-month OS	
	Univariate analysis	Multivariate analysis	Univariate analysis	Multivariate analysis	Univariate analysis	Multivariate analysis
	OR (95% CI)	aOR (95% CI)	OR (95% CI)	aOR (95% CI)	OR (95% CI)	aOR (95% CI)
**Patient-factors**						
Gender						
Male	0.56 (0.25–1.25)		0.61 (0.30–1.24)		0.50 (0.23–1.09)	
Age ≥ 65 years	0.67 (0.25–1.75)		0.55 (0.20–1.53)		0.47 (0.13–1.71)	
Preoperative KPS ≥ 80	1.57 (0.73–3.36)		0.89 (0.43–1.85)		0.91 (0.40–2.08)	
Post-concomitant CRT KPS ≥ 80	1.12 (0.89–2.98)		0.90 (0.41–1.71)		0.98 (0.56–1.99)	
**Tumor-factors**						
Location						
Frontal	0.13 (0.84–4.11)		*1.75 (0.87–3.57)*		*1.91 (0.88–4.17)*	
Temporal	0.51 (0.38–1.62)		*1.34 (0.68–2.67)*		*1.21 (0.56–2.62)*	
Parietal	0.31 (0.22–1.61)		*0.33 (0.10–1.04)*		*0.51 (0.14–1.85)*	
Occipital	0.52 (0.34–8.45)		*0.68 (0.63–2.83)*		*0.35 (0.04–2.87)*	
Insula	0.59 (0.12–2.94)		*0.44 (0.11–1.68)*		*0.24 (0.30–1.96)*	
Laterality						
Left hemisphere	0.96 (0.47–1.97)		0.99 (0.50–1.94)		*0.83 (0.39–1.79)*	
*IDH-1* mutant	3.9 (0.47–3.02)		4.7 (0.92–2.40)		2.98 (1.74–4.65)	
*pMGMT* methylated	2.91 (1.25–6.78)	3.23 (1.33–7.69)	3.16 (1.48–6.75)	3.70 (1.64–8.33)	5.02 (2.06–12.24)	5.88 (1.96–17.24)
**Treatment-factors**						
Extent of resection*						
Class 1:						
supramaximal CE resection i.e. residual tumor:	1.44 (0.89–1.76)		1.34 (0.65–1.97)		1.08 (0.91–1.14)	
0cm^3^ CE + ≤ 5cm^3^ nCE						
Class 2						
maximal CE resection						
A: complete CE resection	1.15 (0.78–1.23)		1.14 (0.78–1.42)		1.56 (0.64–2.13)	
i.e. residual tumor:						
0cm^3^ CE + > 5cm^3^ nCE						
B: near total CE resection						
i.e. residual tumor:	0.89 (0.44–1.31)		0.88 (0.60–1.29)		0.87 (0.57–1.31)	
≤ 1 cm^3^ CE						
Post-concomitant CRT residual CE tumor	0.99 (0.67–1.55)		0.81 (0.58–1.45)		0.89 (0.65–1.27)	
AEF	3.88 (1.57–9.56)	4.00 (1.52-10.00)	3.44 (1.66–7.14)	4.17 (1.85-10.00)	3.07 (1.40–6.73)	5.88 (2.03–16.67)

N.B. OS, overall survival; TMZ, temozolomide; CRT, chemoradiotherapy; AEF, alternating electric fields; KPS, Karnofsky performance status, GTR, gross total resection; STR, subtotal resection; *IDH-1*, isocitrate dehydrogenase-1; p*MGMT*, promoter region of methylguanine-methyltransferase; CE, contrast-enhancing; nCE, non-contrast-enhancing

*Absolute residual tumor volume according to RANO *resect* criteria by Karschnia et al. (in *Journal of Neuro-oncology*, 2023)

*Post-hoc* sensitivity analysis was performed to compare OS between matched CRT-alone control group patients (*n* = 94) and the remaining CRT patients that were not selected for propensity score matching (*n* = 312). The matched CRT-alone patients had a significantly longer mOS of 14.2 months (IQR: 11.3–17.2) compared to those that were excluded from the analysis that had a mOS of 12.3 months (IQR: 10.6–14.0, Cox-regression, *p*-value: 0.03). A subgroup analysis was performed between CRT-alone study control group patients that had preceding gross total tumor resection (41%, 39/94) and their non-selected counterparts (37%, 116/ 312). There was no significant difference in the proportion of patients that had gross total resection (*p*-value: 0.87) between these two groups. The mOS also continued to be significantly shorter for the matched control group patients of this study with a mOS of 14.3 (IQR 9.3–20.3) *versus* 12.4 months (IQR: 10.6–23.6, *p*-value: 0.03). The only identified contributing factor for the disparity in mOS between both groups was the significantly higher proportion of selected control patients that had a preoperative KPS *≥* 80 (70% *versus* 51%) and post-CRT KPS *≥* 80 (73% *versus* 49%) (Table 1).

### Predictors for overall survival among AEF patients

**Fig. 4:**
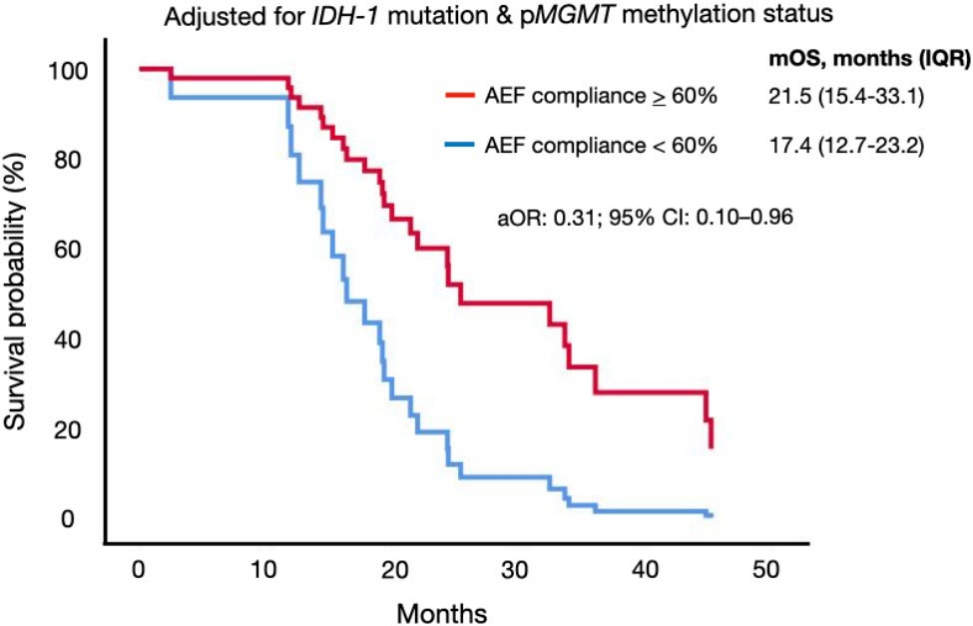
Kaplan-Meier survival analysis demonstrating a mean monthly alternating electric fields therapy compliance *≥* 60% was an independent predictor for overall survival. N.B. AEF, alternating electric fields; *IDH-1*, isocitrate *dehydrogenase-1*, p*MGMT*, promoter region of methylguanine-methyltransferase

AEF was initiated at a mean duration of 43 *±* 28 days after completion of CRT and 161 *±* 147 days (5.4 *±* 4.9 months) after tumor resection. The proportion of patients with a post-concomitant CRT KPS *≥* 80 was 72% (34/47), i.e. before the initiation of AEF. Residual tumor was detected before the initiation of AEF in 55% (26/47) of patients. The mean duration of AEF was 428 *±* 310 days (15.3 *±* 10.3 months) and the mean monthly compliance to treatment was 74 *±* 12%. Age *≥* 65 years, preoperative KPS *≥* 80, post-concomitant CRT KPS *≥* 80, laterality, tumor location, *IDH-1* mutant tumors, p*MGMT* methylated tumors or duration from resection-to-AEF and from CRT-to-AEF were not predictors for survival (Table 3). The only factor associated with longer OS was a monthly mean AEF treatment compliance of *≥* 60% (*≥* 14.5 h per day) (Fig. 4). AEF patients that could achieve this threshold had a mOS of 21.5 months (IQR: 15.4–33.1), conferring a survival benefit of 4.1 months, compared to those that were less compliant (log-rank test, *p*-value: 0.03).

**Table 3:** Predictors for overall survival among AEF + CRT patients

	Univariate analysis	Multivariate analysis
	OR (95% CI)	aOR (95% CI)
**Patient-factors**		
Gender		
Male	2.13 (0.97–4.55)	
Age ≥ 65 years	0.93 (0.36–2.44)	
Preoperative KPS ≥ 80	0.72 (0.32–1.62)	
Post-concomitant CRT KPS ≥ 80	0.78 (0.38–1.79)	
**Tumor-factors**		
*IDH-1* mutant	0.19 (0.03–1.41)	
*pMGMT* methylated	0.67 (0.31–1.43)	
**Treatment-factors**		
Supramaximal or maximal CE	0.63 (0.30–1.32)	
tumor resection*		
Post-concomitant CRT residual CE tumor	0.71 (0.55–1.23)	
CRT-to-AEF ≤ 30 days	0.68 (0.31–1.49)	
Resection-to-AEF ≤ 120 days	0.86 (0.37-2.00)	
AEF compliance ≥ 60%	0.32 (0.10–0.96)	0.31 (0.10–0.96)

N.B. CRT, chemoradiotherapy; AEF, alternating electric fields; KPS, Karnofsky performance status, GTR, gross total resection; *IDH-1*, isocitrate dehydrogenase-1; p*MGMT*, promoter region of methylguanine-methyltransferase

*Absolute residual tumor volume according to RANO *resect* criteria by Karschnia et al. (in *Journal of Neuro-oncology*, 2023)

### AEF-associated adverse effects

The only AEF-associated adverse effect observed was scalp array-induced dermatitis. No systemic adverse reactions or seizures directly attributable to AEF therapy were noted. 77% (36/47) of patients experienced RTOG grade 1 skin toxicity, i.e. maculopapular scalp rash, and 4% (2/47) developed grade 2 toxicity, i.e. dry desquamation. All scalp adverse reactions were completely reversible after temporary AEF therapy cessation (4%, 2/47) of a mean duration of 3.5 *±* 1.6 weeks, or by the application of topical hydrocortisone creams and scalp hydrating emollients. None of the patients had to terminate treatment due to scalp dermatitis.

## Discussion

It has been two decades since the introduction of temozolomide chemoradiotherapy as standard first-line treatment for patients with WHO grade 4 astrocytomas. Although our understanding of the onco-biology of these tumors has advanced, this was not matched by clinically translatable therapeutic breakthroughs. In recent years, AEF has increasingly gained prominence as a novel therapeutic modality that exploits the high dipole moments of tumor cell microtubule substrate proteins tubulin and septin to elicit an anti-mitotic effect [4–6]. The regional application of alternating electric fields disrupts microtubule spindle formation during the M phase of tumor cell division resulting in post-mitotic cellular stress ultimately triggering apoptosis as soon as 24 h after AEF exposure [4, 5]. There is also evidence to suggest that AEF also causes immunogenic cytotoxic effects independent of its anti-mitotic activity. Xenograft animal models revealed that tumor cells exposed to AEF evoked the expression of proinflammatory cytokines such as IFN-β, induced dendritic cell maturation and leukocyte recruitment resulting in extensive intra-tumoral immune cell infiltration [31–33]. This mechanism of action is also supported clinically since delayed glioblastoma regression is frequently observed six to ten months after starting AEF therapy and this deferred oncologic effect is consistent with immune-mediated cell death [34]. 

As global approvals for AEF by healthcare regulatory agencies rise, there is a need for real-world evidence to justify its provision, especially when the financial costs for this therapy are considerable [35]. Ever since the EF-14 RCT concluded the effectiveness of AEF + CRT for newly-diagnosed glioblastoma, six independent real-world cohort studies attempted to validate its therapeutic role [6, 12, 13, 15–17, 19]. All utilised conventional adjusted regression modelling methods to identify control group subjects and the overwhelming majority were single-institution studies with one describing 6-monthly survival rates instead of OS duration [12]. Five of the six studies reported a significant mOS survival benefit for AEF + CRT patients ranging from 5.7 to 6.9 months [13, 15–17, 19]. A subsequent meta-analysis of these independent post-approval studies demonstrated a pooled improvement in mOS of 5.2 months (22.6 months *versus* 17.4 months), but also remarked notable differences between AEF + CRT and control group patients across several prognostic factors in particular with regard to age, p*MGMT* methylation status and EOR [11]. Our review was not only a multi-center study, but also adopted a relatively more rigorous control cohort selection process by propensity score matching. Our observations corroborate previous findings where OS was increased by 7.0 months and after adjusting for *IDH-1* mutation and p*MGMT* methylation, was further extended to 8.5 months. Propensity-score analysis served to reduce selection bias by combining multiple covariates into a single score to control for confounders and accounted for the conditional probability of AEF treatment selection. This quasi-experimental observational study design was adopted since we had access to a comprehensive clinically-annotated central glioma registry with which we could review real-world data [1, 36]. One principal strength of using such data is its reflection of routine patient care, covering a broad spectrum of the patient population, offering greater generalizability and external validation of clinical trial findings [37]. Using propensity-score analysis has gained increasing popularity in the last decade for cancer intervention research with the wider accessibility of databases such as the Surveillance, Epidemiology, and End Results-Medicare (SEER-Medicare) and the National Cancer Data Base (NCDB) [38]. 

Our findings support the conclusions of the EF-14 trial indicating that AEF + CRT confers a significant increase in OS for newly-diagnosed WHO grade 4 astrocytoma regardless of age, functional performance, EOR and p*MGMT* methylation status [6]. A survival benefit in excess of eight months was observed, the longest reported in the biomedical literature and the original EF-14 RCT, a phenomenon seldom observed from real-world studies. Although selection bias may have contributed to this, a review of the unmatched cohorts between the two groups revealed the only significant difference was functional performance. Otherwise, the matched prognostic factor profile of our study patients was comparable to that of a typical WHO grade 4 astrocytoma patient in Hong Kong [1]. These results support the inclusion of AEF as a first-line SOC option for several clinical practice guidelines including the American Society of Clinical Oncology, the Society for Neuro-oncology and the Chinese Brain Cancer Association [39–42]. 

In spite of regulatory approval, considerable scepticism towards AEF therapy exists with fewer than 12% of patients receiving such treatment and among neuro-oncologists, only 30% viewed it as a definitive component of SOC [43]. Several reasons account for this lack of enthusiasm and can be generally classified in relation to the design of the original EF-14 trial, inadequate understanding of the mechanisms of action, perceived effects on quality-of-life (QoL) and the current prohibitive costs of treatment [44, 45]. The EF-14 RCT was an open-labelled study that did not utilize a sham device and its primary endpoint was PFS which can be difficult to determine. Subjects were randomized later in their course at a median interval of 3.8 months after diagnosis and those that experienced rapid progression were excluded (8%, 82/1019). Early randomization is crucial, as the RTOG 0525 RCT observed that a time lag between registration and trial arm assignment resulted in an almost two-month difference in OS [46]. A systematic review of the biomedical literature noted that rapid early progression, defined as post-operative glioblastoma recurrence before the initiation of adjuvant CRT, occurred at a mean incidence of 46% and raises concerns on the generalizability of AEF therapy [47]. A number of preclinical studies have described the mechanistic effects of AEF at the cellular level, but there is a lack of understanding of its influence on the tumor microenvironment. Since therapy requires patients to undergo regular full scalp shaving, treatment for a substantial portion of the day and being connected to a cumbersome device, perceived detrimental concerns for QoL may have dissuaded clinicians, patients and their caregivers. Nevertheless, several studies have confirmed that health-related QoL assessments were not adversely affected by the addition of AEF therapy and could even improve as patients survive longer [48–50]. 

The cost of AEF therapy is a prominent barrier to adoption and there is a general reluctance to bear the heavy financial burdens of this non-curative treatment in exchange for a modest increase in survival. Four studies have evaluated the cost-effectiveness of AEF therapy reviewing patient cohorts managed in the US, France and China [51–54]. Largely dependent on the willingness-to-pay threshold and the incremental cost-effectiveness ratio that varies with each country, two French studies concluded that AEF therapy was not cost-effective, but the remaining studies observed otherwise [51, 53]. Determining the value of anti-cancer therapies especially for uncommon tumors such as glioblastoma is complex and requires a degree of flexible fiscal jurisprudence. For example, although acknowledging that TMZ CRT is not cost-effective, health systems of low-and middle-income countries continue to routinely prescribe standard treatment [55, 56]. Regulatory authorities not only rely on the highest level of evidence offered by RCTs, but also frequently refer to real-world study evidence to evaluate treatment feasibility for broader patient populations and individual societal expectations. Value frameworks, proposed by the American Society of Oncology and the European Society for Medical Oncology respectively, were introduced to better inform policymakers on public healthcare budgeting decisions [57, 58]. Such frameworks are multi-faceted assessments that evaluate net health benefits by reviewing event-free survival, QoL and treatment toxicity for the purpose of ranking the clinically meaningful benefits of novel anti-cancer therapies. Comparable reviews led to national insurance programs reimbursing AEF therapy for selected patients in the US, Japan, Austria, Germany, France, Sweden and Israel. Given the rarity of glioblastoma in Hong Kong with a stable incidence of 1: 100 000 adult population, after a similarly rigorous evaluation of the impact AEF therapy on local patient survival and its adverse effects, the Hong Kong government eventually agreed to subsidize treatment for newly-diagnosed patients [1]. As competing medical products come to market and as the technology continues to evolve, such as implantable intracranial AEF or oncomagnetic devices that are founded on similar therapeutic principles, it is anticipated that the cost of AEF will decrease with time [59, 60]. 

Our findings support prior analyses that demonstrated a dose-response relationship between AEF treatment and OS [6, 15, 61]. A review of field intensities (V/cm) and power densities (mW/cm^3^), utilizing computational dosimetry modelling from EF-14 trial subjects, detected positive correlations with survival [61]. The original trial concluded that a mean monthly compliance, i.e. device usage time, of *≥* 75% was associated with a significant improvement in survival and this extended to elderly patients of *≥* 65 years [6, 62]. This observation was validated by only two independent post-approval studies, but their designs dichotomized treatment compliance groups using a 75% cut-off threshold [15, 18]. A further in-depth analysis of EF-14 trial device usage durations identified that a minimum threshold of 50% resulted in longer OS [63]. We determined that a threshold of 60% AEF treatment compliance was an independent predictor for survival and suggests that lower cut-off device usage durations can still be beneficial.

Due to the nature of long-term AEF array application, it is understandable that a substantial proportion of patients experienced scalp dermatitis with studies reporting an incidence of 25–53%, the majority being RTOG grade 1–2 reactions [18, 62, 64]. Although this was the only adverse effect observed, its occurrence was considerably higher than previously reported. As a south-eastern Chinese coastal city, Hong Kong has a subtropical climate with warm humid weather for most of the year. This could have accounted for the notably higher proportion of patients (77%) that had this adverse effect. Vigilance for the occurrence of these dermatologic reactions and their timely management include the use of topical corticosteroids, oral antipruritic medication or the temporary cessation of AEF treatment [65]. For patients residing in tropical climates, additional mitigating strategies include applying topical aluminium chloride or glycopyrrolate antiperspirants, trimming scalp electrode adhesive tape, applying moist cold compresses to affected areas as well as installing home air-conditioners and dehumidifiers [65]. 

A number of study limitations were identified. As this was a non-randomized study, the risks of bias and the overestimation of treatment effects exist. However, since all data was prospectively collected, that real-world multi-center observations were made and propensity-score matching utilized, it was believed to be the only study design approach to validate AEF effectiveness short of performing a RCT. Several sources of selection bias exist. Patients older than 70 years were not reviewed, but they constitute 18% of glioblastoma patients in the territory and it is expected to be higher as Hong Kong’s population increasingly ages [1]. This was largely due to local Hong Kong neuro-oncologist practice to refrain from administering chemotherapy for older patients as a result of relatively unclear clinical evidence [1]. Two prospective trials and one RCT of elderly glioblastoma patients that compared upfront TMZ alone against RT alone, concluded that chemotherapy was detrimental for those with p*MGMT*-unmethylated tumors without conferring a demonstrable improvement in OS [66–68]. There are reasons to believe that AEF therapy should be offered to older patients. The EF-14 RCT recruited patients as old as 83 years old and a subgroup analysis of patients *≥* 65 years concluded that AEF therapy continued to offer a significant 3.7-month mOS benefit [6, 62]. Bias was also evident in the selection of control group subjects since patients that were not matched and excluded from the analysis had poorer preoperative and post-concomitant CRT functional performance. Another source of bias was the exclusion of patients that underwent rapid progression during or after CRT from receiving AEF therapy. Control group patients were selected from a registry that spans from 2009 to 2023 while those that received additional AEF therapy were treated since 2019. This may have resulted in the possible introduction of confounding management discrepancies over this time period that influenced survival. But since there was no substantial change in TMZ CRT standard-of-care therapy in the intervening years, along with the absence of any novel interventions or clinical trials in Hong Kong during this time, it was believed that the impact of this issue was minimal. A proportion of EOR data was retrieved from operative records reflecting a neurosurgeon’s assessment and not by independent evaluations by early postoperative MRI. The major reason why we relied on such assessments was because of the absence of standard imaging protocols in Hong Kong where only two of the seven neurosurgical centres offer early postoperative scanning. Having neurosurgeons report on their perceived EOR assessment is known to be unreliable and could have contributed to the shorter OS in the control group [69]. The Hong Kong Glioma Registry, a population-based database, did not routinely document QoL and patient-reported outcomes measures data. These assessments would have provided a more nuanced assessment on AEF treatment tolerability. Due to the real-world nature of this study, another limitation was that patients were diagnosed according to the 4th WHO classification [25]. The latest 5th edition recently refined the diagnosis of glioblastoma by adopting a multi-layered integrated approach incorporating new molecular criteria such as *TERT* promoter mutation, *EGFR* amplification or chromosomal 7 + gain / chromosomal 10- loss for *IDH-1* wildtype tumors. It would have been interesting to determine whether certain patient subgroups diagnosed with “molecularly-defined” glioblastoma would have been more responsive to AEF therapy [16]. Due to resource limitations, central governmental funding to perform these molecular tests was only made available in our region in 2024 and could not be retro-actively utilized for archived historical control group tumor tissue. As the EF-14 RCT and other subsequent studies reviewed patients according to the previous 4th WHO classification, we believed that the potential impact of AEF treatment on patient survival in light of the updated definitions would have been minimal. Future research should focus on reviewing the impact of AEF therapy in patients with unfavorable survival prognostic factors such as the elderly, those with poorer functional performance or had rapid progression with an emphasis on assessing health-related QoL.

In conclusion, this first propensity-scored matched prospective multi-center study observed significantly longer OS for newly-diagnosed WHO grade 4 astrocytoma patients that received AEF treatment with TMZ CRT. Regardless of age, functional performance, *IDH-1* mutation, p*MGMT* methylation status or EOR, AEF treatment was an independent predictor for survival. Our findings support the inclusion of such treatment as first-line standard-of-care.

## Data Availability

No datasets were generated or analysed during the current study.

## Abbreviations

AEF: Alternating electric fields
aHR: Adjusted hazard ratio
ANOVA: Analysis of variance
CI: Confidence interval
CNS: Central nervous system
CRT: Chemoradiotherapy
EOR: Extent of resection
GTR: Gross total resection
HA: Hospital Authority
*IDH-1*: Isocitrate dehydrogenase-1
IQR: Interquartile range
KPS: Karnofsky Performance Status
MRI: Magnetic resonance imaging
NCDB: National Cancer Data Base
OS: Overall survival
PFS: Progression-free survival
p*MGMT*: Methylguanine-methyltransferase promoter
QoL: Quality-of-life
RCT: Randomized-controlled trial
RTOG: Radiation Therapy Oncology Group
SD: Standard deviation
SNO: Society for Neuro-oncology
SOC: Standard-of-care
STR: Subtotal resection
TMZ: Temozolomide
WHO: World Health Organization
